# Serum uric acid to HDL-Chol ratio (UHR) is associated with insulin resistance/sensitivity in individuals without diabetes

**DOI:** 10.1007/s00592-025-02576-2

**Published:** 2025-08-27

**Authors:** Mariangela Rubino, Mattia Massimino, Elettra Mancuso, Carolina Averta, Angela Palummo, Maria Perticone, Elena Succurro, Angela Sciacqua, Gaia Chiara Mannino, Francesco Andreozzi

**Affiliations:** 1 Department of Medical and Surgical Sciences, University Magna Graecia of Catanzaro, Catanzaro, 88100 Italy; 2 Department of Health Sciences, University Magna Graecia of Catanzaro, Catanzaro, 88100 Italy; 3 Department of Experimental and Clinical Medicine, University Magna Graecia of Catanzaro, Catanzaro, 88100 Italy

**Keywords:** Uric acid-to-HDL cholesterol ratio, Insulin resistance, Low grade inflammation, Hyperinsulinemic-euglycemic clamp, Prediabetes, Type 2 diabetes mellitus

## Abstract

**Background:**

The uric acid-to-HDL cholesterol ratio (UHR) is a promising non-insulin-based marker for metabolic risk, associated with type 2 diabetes, hypertension, hepatic steatosis, and cardiovascular disease. However, its utility in individuals with altered glucose tolerance remains unclear.

**Methods:**

We investigated the relationship between UHR and insulin sensitivity in two independent cohorts. Sample 1 (*n* = 1555) from the CATAMERI study, was stratified based on oral glucose tolerance test (OGTT) results, and Sample 2 (*n* = 332) from the EUGENE2 project, with insulin sensitivity measured via euglycemic-hyperinsulinemic clamp.

**Results:**

In Sample 1, UHR showed positive correlations with BMI, triglycerides, 2-hour plasma glucose, HOMA-IR, fasting plasma insulin (*p* < 0.0001 for all) and with HbA1c (*p* < 0.001), and negative correlations with Matsuda index (*p* < 0.0001) and total cholesterol (*p* = 0.019). Multivariable linear regression identified HOMA-IR (β = 0.100), Matsuda index (β=-0.146), InsAUC30/GluAUC30 (β = 0.120), and Stumvoll 1st-phase insulin secretion (β = 0.121) as independent UHR predictors. In Sample 2, bivariate analyses, adjusted for age, sex, and BMI, confirmed positive correlations between UHR and HbA1c (*p* < 0.001), 2-hour post-load glucose (*p* = 0.001), BMI, triglycerides, and fasting insulin (*p* < 0.0001 for all) and a negative correlation with Clamp M (glucose disposal, *p* = 0.0003). Finally, multivariable regression of Clamp M variability (adjusted for age, sex, and BMI) demonstrated significant negative associations with UHR (β= -0.230) and BMI (β= -0.375).

**Conclusion:**

These findings suggest that UHR, derived easily and inexpensively from routine clinical measurements, is a promising indicator of metabolic risk in individuals without diabetes. Its accessibility positions it as a potential tool for early diabetes prevention strategies, potentially reducing reliance on the OGTT.

**Supplementary Information:**

The online version contains supplementary material available at 10.1007/s00592-025-02576-2.

## Introduction

Type 2 Diabetes Mellitus (T2DM) is a chronic disease characterized by hyperglycemia due to a combination of reduced insulin secretion and resistance of peripheral tissues to insulin action [1]. Recent epidemiological data estimate that more than 500 million people, aged between 20 and 80, are affected by diabetes with a significant impact on medical costs and health worldwide [2]. Similar figures describe the prevalence of prediabetes [2], a condition defined by specific diagnostic criteria [3] and associated with increased risk of developing T2DM.

The identification of high-risk categories for developing T2DM has proved to be a winning strategy as it allows for the early identification of those who would benefit most from close monitoring, lifestyle, diet and physical activity interventions.

In recent years there has been an increase in the number of non-insulin-based indices of insulin resistance that have been proposed as predictors of T2DM incidence [4–6]. Among them, we have focused our attention on the uric acid (UA)-to- high density lipoprotein cholesterol (HDL-Chol) ratio (UHR), the most powerful predictor of metabolic deterioration [7], which combines circulating levels of UA and HDL-Chol. Both parameters are commonly screened in clinical practice and are highly informative for the physician in assessing the conditions of a patient. HDL-Chol plays important physiological roles, it mediates reverse cholesterol transport, recruiting and conveying excess peripheral cholesterol to the liver, where it is metabolized. Low levels of HDL-Chol are associated with increased risk of atherosclerotic cardiovascular disease (CVD) [8]. UA is the final product of purine metabolism [9] and an increase in this metabolite is frequently found in insulin resistance, hypertension and reduced renal function [10–12].

Recent studies have shown that high levels of UHR are associated with diseases characterized by low-grade chronic inflammation such as non-alcoholic fatty liver disease, diabetic nephropathy, hypertension and Hashimoto’s thyroiditis in addition to T2DM [13–16]. Moreover, it is known that elevated UHR levels are associated with reduced insulin sensitivity, metabolic syndrome and predict the development of T2DM [7]. Zhou et al. [17] have recently reported that UHR is associated with the risk of insulin resistance in a general population extracted from the National Health and Nutrition Examination Survey (NHANES). Insulin resistance is a feature shared by all the metabolic conditions and inflammatory disorders to which UHR is associated, nevertheless, there is little evidence of the specific relationship between UHR and parameters of insulin resistance/sensitivity.

In the present study we aimed to address this issue by taking advantage of a large cohort of metabolically well-characterized European subjects without diabetes, with different degrees of glucose tolerance. In this cohort, surrogate indices of pancreatic β-cell function have been derived from the 75 g-oral glucose tolerance test (OGTT), and the gold standard technique hyperinsulinemic-euglycemic clamp has been employed to determine peripheral insulin sensitivity [18].

## Methods

### Data availability statement

All data generated or analysed during this study are included in this published article.

### Study subjects

Two independent samples of adult (≥ 18 years of age) individuals without diabetes of European ancestry were studied. Sample 1 comprised 1555 self-reported Caucasian subjects without diabetes, enrolled in the CAtanzaro MEtabolic RIsk factors Study (CATAMERIS) [19], who underwent a 75 g OGTT with 0, 30, 60, 90 and 120 min sampling for the assessment of plasma glucose and plasma insulin levels. Sample 2 includes 332 individuals from the European Network on Functional Genomics of Type 2 Diabetes (EUGENE2) project, for whom insulin sensitivity was assessed by euglycemic-hyperinsulinemic clamp, as previously described [20]. Briefly, a priming dose of insulin (Humulin, Eli Lilly & Co., Indianapolis, IN) was administrated during the initial 10 min to acutely raise plasma insulin followed by continuous insulin infusion fixed at 40 mU/m2 x min. The blood glucose level was maintained constant during the 2-h clamp study by infusing 20% glucose at varying rates according to blood glucose measurements assessed by a glucose analyzer at 5-minute intervals (mean coefficient of variation of blood glucose was < 5%). Blood samples for plasma insulin assay were drawn at 60, 80, 100, and 120 min during the clamp study. Both recruited cohorts include individuals consecutively recruited at the Department of Medical and Surgical Sciences of the University “Magna Graecia” of Catanzaro in a campaign for the assessment of cardio-metabolic risk factors. All patients underwent a thorough examination, and anthropometric, biochemical and clinical data were collected at the recruitment visit. Blood sampling for laboratory determinations was performed in the morning after an overnight fasting. The inclusion criteria were fasting plasma glucose (FPG) < 126 mg/dl and presence of one or more cardio-metabolic risk factors including hypertension, dyslipidemia, and overweight/obesity. Subjects were excluded if they had chronic gastrointestinal diseases, chronic pancreatitis, history of any malignant disease, history of alcohol or drug abuse, positivity for antibodies to hepatitis C virus (HCV) or hepatitis B surface antigen (HBsAg), kidney or hepatic failure. The study was approved by the Institutional Ethics Committees of the University “Magna Graecia” of Catanzaro. Written informed consent was obtained from each subject in accordance with the principles of the Declaration of Helsinki.

### Calculations

UHR was calculated as the ratio between serum UA and HDL-Chol levels [21]. The Matsuda Index (also known as insulin sensitivity index, ISI) was calculated as follows: 10,000/square root of (fasting glucose (mmol/L) x fasting insulin (mU/L)) x (mean glucose x mean insulin during OGTT) [22]. The homeostasis-model-assessment-estimated insulin resistance (HOMA-IR) was calculated as fasting insulin x fasting glucose/22.5 [23]. Two indexes of insulin secretion were obtained from the OGTT: the Stumvoll index (1st-phase secretion = 1283 + 1.829 x Ins30 ± 138.7 x Gluc30 + 3.772 x Ins0) [24]; and the ratio of insulin AUC0-30 to glucose AUC0-30 (InsAUC30/GlucAUC30) [25]. In both cases Ins is insulin and Gluc is glucose at the indicated time points, or their area under the curve (AUC). Glucose disposal (Clamp M) was calculated as the mean rate of glucose infusion during the last 60 min of the clamp examination (steady-state) and is expressed in milligrams per minute per kilogram of fat-free mass measured by electrical bioimpedance [26]. Estimated glomerular filtration rate (eGFR) was calculated by the CKD-EPI equation: eGFR = 141 x min(Scr/k,1)^a^ x max(Scr/k, 1)^−1.209^ × 0.993^Age^ x 1.018 [if female], where Scr is serum creatinine, k is 0.7 for females and 0.9 for males, a is -0.329 for females and − 0.411 for males, min indicates the minimum of Scr/k or 1, and max indicates the maximum of (Scr/k or 1) [27].

### Laboratory determinations

HbA1c was measured with high performance liquid chromatography using a National Glycohemoglobin Standardization Program (NGSP) certified automated analyzer (Adams HA-8160 HbA1C analyzer, Menarini, Italy). Triglycerides (TG), total cholesterol (Tot-Chol), and HDL-Chol concentrations were determined by enzymatic methods (Roche, Basel, Switzerland). Low density lipoprotein cholesterol (LDL-Chol) was calculated as the difference between Tot-Chol and HDL-Chol. Plasma glucose was measured by the glucose oxidation method (Beckman Glucose Analyzer II; Beckman Instruments, Milan, Italy). Plasma insulin concentration was measured with a chemiluminescence-based assay (Immulite^®^, Siemens, Italy). Serum creatinine was measured by creatinine Jaffè compensated Method for serum and plasma (Roche Diagnostics). High sensitivity C-reactive protein (CRP) levels were measured by automated instrument BN™II System analyser (Siemens Healthcare, Marburg, Germany) with the CardioPhase^®^ hsCRP kit.

### Statistical analysis

The nature of the study is entirely cross-sectional. The results for continuous variables are given as means ± SD. Categorical variables are presented as percentages. The χ^2^ test was used for categorical variables. Variables with skewed distribution (i.e. TG, fasting insulin, HOMA-IR, and InsAUC30/GluAUC30) were log-transformed to meet the normality assumption for statistical purposes. Cases with randomly missing data were excluded from the analyses following a pairwise approach, based on the variables involved in each test. Correlation coefficients were calculated according to Pearson’s method. Partial correlation coefficients were adjusted for age, sex, and BMI, as indicated. A multivariable stepwise linear regression was used to examine the relation of UHR and the indices of insulin sensitivity (HOMA-IR and Matsuda index) and of insulin secretion (InsAUC30/GluAUC30 and Stumvoll 1st-phase) in a statistical model including several potential confounders as indicated. Areas under the receiver operative characteristic curve (AUROC) were analyzed to determine cutoff values of the UHR index in predicting glucose tolerance status with normal glucose tolerance as the reference category. For all analyses, *p* ≤ 0.05 was considered statistically significant. Given the relatively high number of correlations tested, Bonferroni correction for multiple testing was applied, therefore the threshold for statistical significance was set at *p* < 0.0029 (0.05/17 tests). All analyses were performed using the statistical package SPSS 22.0 for Windows (SPSS, Chicago, IL, USA).

## Results

A total of 1555 individuals were enrolled as Sample 1 and classified according to their glucose tolerance status based on OGTT glucose measurements [3] as having normal glucose tolerance (NGT) when FPG was < 5.6 mmol/L (100 mg/dl) and 2-h post-load < 7.8 mmol/L (140 mg/dl), impaired fasting glucose (IFG) when FPG was 5.6-7.0 mmol/L (100–126 mg/dl) and 2-h post-load < 7.8 mmol/L (140 mg/dl), or impaired glucose tolerance (IGT) when FPG was < 7.0 mmol/L (126 mg/dl) and 2-h post-load was 7.8–11.0 mmol/L (140–199 mg/dl). Overall, 897 were classified as NGT and 658 as IFG/IGT.

In Table 1 the summary data for the two tolerance groups are reported. NGT subjects were younger (*p* < 0.0001), had a higher prevalence of females (*p* < 0.0001) and a lower BMI (*p* < 0.0001), and showed a significantly better profile for metabolic parameters, such as FPG, 2-h post load plasma glucose, insulin levels and HbA1c, (each with *p* < 0.0001). No significant differences were reported for LDL-Chol and Tot-Chol levels, whereas HDL-Chol was significantly higher in the NGT group (*p* < 0.0001), and TG were significantly higher in the IFG/IGT group (*p* < 0.0001). Systolic and diastolic blood pressure appeared significantly lower in the NGT group compared to IFG/IGT (*p* < 0.0001 and *p* < 0.001, respectively). A greater proportion of IFG/IGT individuals had hyperlipidemia and hypertension; eGFR was normal (> 90 ml/min/1.73m^2^), but higher in the NGT group. Circulating CRP concentrations, employed as a marker of subclinical inflammation, were significantly higher in individuals with prediabetes (*p* = 0.015).

**Table 1:** Anthropometric and metabolic characteristics of the sample 1 stratified according to the glucose tolerance

Variables	NGT (*N* = 897)	IFG/IGT (*N* = 658)	*P*
Sex (M/F) %	38/62	55/45	0.015
Age (years)	44 (± 14)	54 (± 12)	< 0.0001
BMI (Kg/m^2^)	29.9 (± 7.0)	31.6 (± 6.9)	< 0.0001
SBP (mmHg)	122.2 (± 15.8)	130.3 (± 16.5)	< 0.0001
DBP (mmHg)	77.1 (± 10.9)	79.2 (± 10.7)	< 0.001
Tot-Chol (mg/dl)	194.4 (± 37.7)	197.8 (± 37.8)	0.154
HDL-Chol (mg/dl)	52.7 (± 14.3)	48.6 (± 12.3)	< 0.0001
LDL-Chol (mg/dl)	124.3 (± 33.6)	126.9 (± 32.9)	0.360
Triglycerides (mg/dl)	111.2 (± 60.8)	139.9 (± 74.0)	< 0.0001
UA (mg/dl)	4.84 (± 1.30)	5.48 (± 1.36)	< 0.0001
UHR	9.99 (± 4.39)	12.07 (± 4.89)	< 0.0001
eGFR (ml/min/1.73m^2^)	136.7 (± 40.1)	117.3 (± 37.3)	< 0.0001
CRP (mg/l)	3.68 (± 4.58)	4.37 (± 6.16)	0.015
FPG (mg/dl)	87.1 (± 7.3)	100.3 (± 10.5)	< 0.0001
2-h PG (mg/dl)	106.2 (± 20.4)	148.9 (± 27.4)	< 0.0001
FPI (U/l)	12.9 (± 8.8)	15.8 (± 9.9)	< 0.0001
HbA1c mmol/mol (%)	36 ± 4 (5.4 ± 0.33)	39 ± 5 (5.71 ± 0.38)	< 0.0001
HOMA-IR	2.76 (± 2.01)	3.80 (± 2.39)	< 0.0001
Matsuda Index	4.53 (± 2.64)	2.89 (± 1.73)	< 0.0001
Stumvoll 1st-phase Index	1764.9 (± 997.6)	1506.5 (± 922.5	< 0.0001
InsAUC30/GluAUC30	8.59 (± 5.75)	6.91 (± 4.47)	< 0.0001
Hypolipidemic Therapy %	23.7%	31.2	0.001
Hypotensive Therapy %	36.2%	60.2	< 0.001
Diuretics	11.5%	25.1%	< 0.0001
ARBs	27.1%	49.2%	< 0.0001
Smokers %	41	48	0.319
Current	24	17	0.220
Ex	18	31	0.03

The data are presented as means ± SD for continuous variables and number (percentages) for dichotomous variables. P values for comparisons between two groups were obtainled using unpaired Student’s t-test or χ^2^ test as appropriate

* ARB* angiotensin receptor blockers, *BMI* body mass index, *CRP* C-Reactive protein, *DBP* diastolic blood pressure, *eGFR* Estimated Glomerular Filtration Rate, *FPG*, Fasting Plasma Glucose, *FPI* Fasting Plasma Insulin, *2-h PG* 2 h-plasma glucose, *HbA1c*, Haemoglobin A1c, *HDL-Chol* high-density lipoprotein cholesterol, *HOMA-IR*, homeostasis model assessment index of insulin resistance, *LDL-Chol* low density lipoprotein, *SBP* systolic blood pressure, *UA* uric acid, *UHR* uric acid-to- HDL-Chol ratio, *Tot-Chol* Total cholesterol

As expected, the HOMA-IR was significantly higher in the IFG/IGT group (*p* < 0were significantlytsuda index was higher in the NGT group (*p* < 0.0001). Consistent with this, the indices of insulin secretion (Stumvoll 1st-phase and InsAUC30/GluAUC30) were significantly reduced in IFG/IGT subjects (*p* < 0.0001 for both parameters). Finally, the UHR was higher in the IFG/IGT group compared to the NGT group (*p* < 0.0001).

We examined the partial correlation between UHR and the other parameters, and the results are reported in Table 2 (adjusted for age, sex and BMI as appropriate). UHR correlated positively with BMI (*r* = 0.310, *p* < 0.0001), TG (*r* = 0.400, *p* < 0.0001), CRP (*r* = 0.115, *p* < 0.0001), HbA1c (*r* = 0.117, *p* < 0.001), 2-h plasma glucose (*r* = 0.079, *p* < 0.0001) and fasting plasma insulin (FPI) (*r* = 0.170, *p* < 0.0001). We found a negative correlation only with the Matsuda index and eGFR (*r*= -0.229, *p* < 0.0001 and *r*= -0.206, *p* < 0.0001, respectively).

**Table 2:** Partial correlations between UHR and metabolic variables

Variables	Pearson’s correlation coefficient (*r*)	*P*
Age (years)	0.027	0.284*
BMI (Kg/m^2^)	0.310	< 0.0001**
SBP (mg/dl)	0.026	0.314
DBP (mmHg)	0.018	0.497
Tot-Chol (mmHg)	-0.060	0.019
LDL-Chol (mg/dl)	0.020	0.437
Triglycerides (mg/dl)	0.400	< 0.0001
eGFR	-0.206	< 0.0001
CRP (mg/L)	0.115	< 0.0001
HbA1c	0.117	< 0.001
FPG (mg/dl)	0.079	0.002
2-h PG (mg/dl)	0.151	< 0.0001
FPI (mU/ml)	0.170	< 0.0001
HOMA-IR	0.177	< 0.0001
Matsuda Index	-0.229	< 0.001
Stumvoll 1st-phase Index	0.154	< 0.001
InsAUC30/GluAUC30	0.144	< 0.001

P values of partial correlation coefficients are adjusted for age, sex and BMI. * P values refer to results after analyses with adjustment for sex and BMI. ** P values refer to results after adjustment for sex and age. A p-value < 0.0029 (0.05/17[number of tests]) is considered statistically significant

*MI* body mass index, *CRP* C-Reactive protein, *DBP* diastolic blood pressure, *eGFR* Estimated Glomerular Filtration Rate, *2-h PG* 2 h-plasma glucose, *FPG* Fasting Plasma Glucose, *FPI* Fasting Plasma insulin, *HbA1c* Haemoglobin A1c, *HOMA-IR* homeostasis model assessment index of insulin resistance, *LDL-Chol* low density lipoprotein, *SBP* systolic blood pressure, *Tot-Chol* Total cholesterol;

To evaluate the independent contribution of UHR to the variability in the indices of insulin sensitivity and secretion, we performed a multivariable regression analysis, adjusted for the variables significantly correlated with UHR (Tables 3 and 4). Because insulin sensitivity and secretion are different according to glucose tolerance, we also included glucose tolerance status (NGT, IFG, IGT, or IFG + IGT) among the potential confounders. Comparison of standardized coefficients allowed the determination of the relative strength of the association of UHR with each dependent variable. We found that UHR was associated significantly with HOMA-IR (β = 0.101, *p* = 0.007, Table 3), Matsuda index (β = -0.120, *p* < 0.001, Table 3), InsAUC30/GluAUC30 (β = 0.130, *p* = 0.001, Table 4) and Stumvoll 1st-phase (β = 0.130, *p* = 0.001, Table 4). Unstandardized coefficients and 95% CI are provide as supplementary material (Suppl. S1). No differences were observed when the use of dyslipidemia therapy, presence of hypertension, and use of diuretics were included in the analysis (data not shown).

**Table 3:** Stepwise multivariable regression analysis of HOMA-IR and Matsuda index as dependent variable and different covariates, in sample 1

Dependent variable	Independent contributors	Standardized coefficent β	*p*
HOMA-IR	UHR	0.101	0.007
	BMI	0.339	< 0.0001
	TG	0.122	< 0.0001
	Age	-0.093	0.01
	HbA1c	0.068	0.037
	GTS	0.174	< 0.0001
Matsuda Index	UHR	-0.120	0.001
	BMI	-0.315	< 0.0001
	TG	-0.179	< 0.0001
	GTS	-0.207	< 0.0001

Data represent effect sizes (β standardized coefficients) per unit increase and corresponding *p* values obtained when the analyses were performed in Sample 1

*BMI* body mass index, *TG* triglycerides, *HbA1c* Haemoglobin A1c, *HOMA-IR* homeostasis model assessment index of insulin resistance, *GTS *Glucose Tolerance Status

**Table 4:** Multiple regression analysis of InsAUC30/GluAUC30 and Stumvoll 1st-phase index as dependent variables and different covariates, in sample 1

Dependent variable	Independent contributors	Standardized coefficent β	*p*
InsAUC30/GluAUC30	UHR	0.130	0.001
	BMI	0.245	< 0.0001
	TG	0.101	0.002
	Age	-0.202	< 0.0001
	GTS	-0.166	< 0.0001
Stumvoll 1st-phase Index	UHR	0.130	0.001
	BMI	0.276	< 0.0001
	TG	0.111	0.001
	Age	-0.200	< 0.0001
	GTS	-0.151	< 0.0001

Data represent effect sizes (β standardized coefficients) per unit increase and corresponding *p* values obtained when the analyses were performed in Sample 1

*BMI* body mass index, *TG* triglycerides, *HbA1c* Haemoglobin A1c, *GTS* Glucose Tolerance Status

To compare the predictive value of UHR to the other indeces, we measured the AUROCs for discriminating the NGT group from individuals with prediabetes. In the study population, the AUROCs for UHR and HOMA-IR were 0.63 (95% CI: 0.60–0.66, *p* < 0.0001) and 0.67 (95% CI: 0.64–0.70, *p* < 0.0001), respectively (Supplementary Fig. 1), and the optimal cut-offs for UHR and HOMA-IR were 8.63 (sensitivity: 75.8%, specificity: 44.8%) and 2.95 (sensitivity: 59.5%, specificity: 68.2%). The discriminatory power of the two markers was almost identical and relatively small (0.60 < AUROC < 0.70). The AUROCs for Matsuda index, InsAUC30/GluAUC30 and Stumvoll 1st-phase were small (0.29, 0.41, and 0.40, respectively) and had no discriminatory value (AUROC < 0.50).In order to get further insights on the association of UHR on insulin sensitivity, we analyzed an additional sample of 332 individuals without diabetes (Sample 2) who undertook the assessment of insulin-stimulated glucose disposal (Clamp M) by euglycemic hyperinsulinemic clamp. The summary statistics of Sample 2 were similar to those reported for Sample 1 (Suppl. S2) and the NGT group exhibited a better metabolic profile. The univariate analyses adjusted for age, sex and BMI, confirmed the positive significant correlation of UHR with BMI (*r* = 0.459, *p* < 0.0001), TG (*r* = 0.313, *p* < 0.0001), HbA1c (*r* = 0.217, *p* < 0.01), 2-h post-load glucose (*r* = 0.186, *p* = 0.001) and FPI (*r* = 0.255, *p* < 0.0001), while revealing a negative correlation only with Clamp M (*r*= -0.197, *p* = 0.0003).

Finally, to estimate the contribution of UHR to insulin sensitivity measured as glucose disposition index, we performed a multivariable regression analysis adjusted for age, sex, BMI. The results showed a significant negative association of UHR (β = -0.230, *p* < 0.0001) and BMI (β = -0.375, *p* < 0.0001) on the Clamp M variability.

## Discussion

Recently, several indices of insulin sensitivity have been examined, among which UHR, a novel inflammatory and metabolic marker associated with the onset of T2DM and hypertension, hepatic steatosis, and CVD [7, 13, 15, 28].

UA is generated by the liver and represents the last metabolite of purine catabolism [11]. It is known that UA in the normal range can behave as a mild anti-oxidant [29], conversely, higher levels are associated with a broad spectrum of diseases such as diabetes, CVD, hypertension and metabolic syndrome [15, 30–32]. A comprehensive meta-analysis reported that each 1 mg/dL increase in UA conferred about 13% higher risk of developing T2DM, independently of age, sex and other metabolic risk factors. Consistently, insulin resistance tended to worsen as UA levels rose, reinforcing the epidemiological link between hyperuricemia and impaired insulin sensitivity [33]. Accumulating evidence suggests that excess UA can actively contribute to insulin resistance via pro-inflammatory and oxidative pathways. Recently we reported that hyperuricemia might induce inflammation at the hepatocellular level by triggering intracellular oxidative stress and activating proinflammatory NF-κB and Nucleotide-binding domain, leucine-rich repeat, pyrin domain containing 3 (NLRP3) inflammasome signaling cascades, playing an important pathogenetic role in metabolic disorders and CVD [34]. Indeed, markers of low-grade inflammation and others have proven to be useful to detect long-term metabolic risk in people with intermediate diabetes [35–40]. Our research group has further elucidated a novel, oxidative-stress-independent, mechanism whereby UA promotes insulin resistance; in human umbilical vein endothelial cells (HUVECs)by enhancing the interaction between ectonucleotide pyrophosphatase/phosphodiesterase-1 (ENPP1) and the insulin receptor. This augmented ENPP1 binding inhibits receptor autophosphorylation and downstream signalling, directly blunting cellular responsiveness to insulin and providing a new molecular model for UA-induced insulin resistance [41]. These molecular effects support hyperuricemia role as more than a bystander, instead acting as a pathogenic factor in insulin resistance and cardiometabolic disease.

HDL-Chol is globally considered as one of the five components of metabolic syndrome which is associated with chronic conditions such as CVD, T2DM and hypertension [42], and low circulating HDL-Chol levels have been reported as an independent risk factor for cardiovascular damage [43]. Converging clinical and mechanistic data indicate that inadequate HDL-Chol quantity or function actively promotes insulin resistance [44]. Reduced HDL-Chol levels predict incident T2DM and worsening glycaemic control, independently of classical risk factors [45, 46]. Some reports [47–49], have shown that HDL particles and ApoA-1, the main protein component of HDL, exert their beneficial effect on glucose homeostasis by stimulating insulin secretion and β-cell function [50]. At the cellular level, HDL safeguards β-cell integrity by exporting excess cholesterol via the ABCA1/ABCG1 transporters; whereas loss of this efflux pathway leads to lipid accumulation, inflammation, and impaired glucose-stimulated insulin secretion, hastening β-cell dysfunction [51, 52]. Similarly, our previous studies demonstrated a direct effect of HDL particles on α-cells function, in vivo and in vitro [53]. HDL and ApoA-1 were able to inhibit glucagon expression and release upon activation of SCARB-1 catalytic activity, which in turn stimulated the PI3K/Akt/FoxO1 signaling pathway. In the skeletal muscle, ApoA-I binds to ABCA1 and triggers CaMKK1, inducing AMPK signaling cascade that mobilizes GLUT4 to the sarcolemma, permitting insulin-independent glucose uptake [47, 54]. These and other evidences explain, at least in part, the beneficial role played by HDL in glycemic regulation.

To date, there is little evidence of the effectiveness of using UHR in the population with pre-diabetes, i.e. those at highest risk of developing diabetes in the near future. For this reason, we studied the effect of UHR on insulin sensitivity in 2 cohorts with 1887 white European participants without diabetes. In the first group of 1555 patients who underwent an OGTT, we found that UHR was significantly associated with reduced insulin sensitivity assessed by HOMA-IR and Matsuda Index. In the second group, (*N* = 332) we confirmed the association between UHR and insulin sensitivity by using the gold standard technique, euglycemic–hyperinsulinemic clamp method. Indeed, the linear regression analysis demonstrated that UHR was significantly associated with insulin sensitivity (glucose disposal, or Clamp M) regardless of several confounding variables.

Despite our findings of reduced insulin secretion in prediabetes, we observed a positive linear association between UHR and insulin secretion indices (InsAUC30/GluAUC30 and Sturmvoll 1st phase) within our study population. This seemingly paradoxical result could be misinterpreted as a protective effect. However, we contend that this association is not indicative of a beneficial role of UHR, but rather reflects a compensatory physiological mechanism in individuals with a more challenging metabolic profile. We propose that a higher UHR serves as a marker of increased cardiometabolic risk, often correlating with greater peripheral insulin resistance and heightened systemic low-grade inflammation. In this context, the observed elevated insulin secretion represents the β-cell’s strenuous effort to overcome these metabolic challenges and maintain glucose homeostasis, a critical adaptive response [55–57]. Future longitudinal studies, specifically examining changes in insulin secretory function in relation to uric acid and HDL levels across different stages of glucose tolerance, are crucial. Such research could provide deeper insights into the dynamic interplay between these metabolic markers and β-cell function, clarifying the trajectory from compensation to decompensation.

The strength of our report lies in the large-scale sample size, the homogeneity of the patient group, the detailed characterization of the study population, and the centralization of biochemical analyses. Furthermore, the presence of in-study validation in a large independent group of patients subjected to hyperinsulinemic euglycemic clamp supports the reliability and replicability of our results.

Some limitations of the current report should be acknowledged. First, laboratory determinations and OGTT have been performed only once, as per canonical hospital setting, and the hyperinsulinemic euglycemic clamp studies technique is invasive, expensive, time-consuming, and not suitable for large-scale studies, therefore intra-individual variation in insulin sensitivity could not be taken into account. Second, although small differences in insulin sensitivity were found between males and females, the multivariate analysis included all subjects. However, sex differences were considered in the multivariate models. Third, among the several factors able to affect UA levels, we have considered and excluded glomerular filtration rate, use of diuretics and anti-hypertensive therapy, but we cannot estimate the influence of dietary patterns or alcohol consumption (< 20 g/day). Furthermore, due to the cross-sectional setting of our study, the statistical independent associations observed do not imply causality, and the present findings should be considered as hypothesis generating and requiring confirmation by further larger studies. Finally, our findings should not be generalized to other ethnic populations.

Overall, we hereby report that the UHR, which uses values commonly assessed in clinical practice, economical and easily accessible, can be a reference parameter of metabolic risk in individuals without diabetes. As measurements of UA and HDL-Chol levels can be easily obtained through routinary lab examinations and relatively cheap techniques, UHR can be estimated easily in order to implement effective strategies to avoid progression to diabetes, even before requesting the OGTT, which remains an invasive test that is often poorly tolerated by patients.

## Conclusion

The results of our study clearly demonstrate that UHR could be informative in subjects without diabetes and given that its measurement is non-invasive and low-cost, it could become a routine examination, although further prospective studies should confirm the validity of UHR as an additional indicator of impaired glucose metabolism in a wider population of mixed ethnicity.

## Supplementary Information

Below is the link to the electronic supplementary material.

Supplementary Material 1

Supplementary Material 2

Supplementary Material 3

## Data Availability

The authors declare that all data supporting the findings of this study are available from the corresponding author on reasonable request.
