# Supplementary material for: Serum uric acid to HDL-Chol ratio (UHR) is associated with insulin resistance/sensitivity in individuals without diabetes
Source: Acta Diabetol. 2025 Aug 27;63(1):87–95. doi: 10.1007/s00592-025-02576-2 (PMC12847180; doi:10.1007/s00592-025-02576-2)
Supplement: Supplementary file 1 — Supplementary Material 1 [file 592_2025_2576_MOESM1_ESM.docx]

**Suppl. S1. Stepwise multivariable regression analysis of HOMA-IR and Matsuda Index as dependent variable and different covariates, in Sample 1.**

| **Dependent variable** | **Independent Contributors** | **Unstandardized Coefficent** | **95% CI**  **Lower Upper** | |
| --- | --- | --- | --- | --- |
| **HOMA-IR** | **UHR** | **0.046** | **0.013** | **0.079** |
|  | BMI | 0.113 | 0.092 | 0.134 |
|  | TG | 0.004 | 0.002 | 0.006 |
|  | Age | -0.021 | -0.032 | -0.010 |
|  | HbA1c | 0.379 | 0.023 | 0.735 |
|  | GTS | 0.348 | 0.226 | 0.469 |
| **Matsuda Index** | **UHR** | **-0.059** | **-0.944** | **-0.024** |
|  | BMI | -0.114 | -0.136 | -0.092 |
|  | TG | -0.006 | -0.008 | -0.004 |
|  | GTS | -0.452 | -0.580 | -0.324 |

| **Dependent variable** | **Independent Contributors** | **Unstandardized Coefficent** | **95% CI**  **Lower Upper** | |
| --- | --- | --- | --- | --- |
| **InsAUC30/GluAUC30** | **UHR** | **0.140** | **0.056** | **0.223** |
|  | BMI | 0.193 | 0.140 | 0.246 |
|  | TG | 0.007 | 0.003 | 0.012 |
|  | Age | -0.078 | -0.106 | -0.049 |
|  | GTS | -0.789 | -1.095 | -0.483 |
| **Stumvoll 1st-phase Index** | **UHR** | 25.934 | 10.623 | 41.244 |
|  | BMI | 40.327 | 30.615 | 50.039 |
|  | TG | 1.471 | 0.624 | 2.318 |
|  | GTS | -132.91 | -188.98 | -76.83 |

Note: Data represent effect sizes (no standardized coefficients) per unit increase and corresponding *p* values obtained when the analyses were performed in Sample 1. Abbreviations: BMI, body mass index; TG, triglycerides; HbA1c, Haemoglobin A1c; GTS, Glucose Tolerance Status; HOMA-IR, homeostasis model assessment index of insulin resistance.
