# Supplementary material for: Serum uric acid to HDL-Chol ratio (UHR) is associated with insulin resistance/sensitivity in individuals without diabetes
Source: Acta Diabetol. 2025 Aug 27;63(1):87–95. doi: 10.1007/s00592-025-02576-2 (PMC12847180; doi:10.1007/s00592-025-02576-2)
Supplement: Supplementary file 2 — Supplementary Material 2 [file 592_2025_2576_MOESM2_ESM.docx]

**Suppl. S2.** **Anthropometric and metabolic characteristics of the Sample 2 stratified according to the glucose tolerance.**

| **Variables** | **NGT**  **(N=154)** | **IFG/IGT (N=178)** | ***P*** |  |
| --- | --- | --- | --- | --- |
|  |  |  |  |  |
| Gender (M/F) % | 20/80 | 27/73 | 0.243* |  |
| Age (years) | 39 (±10) | 43 (±10) | <0.0001** |  |
| BMI (Kg/m^2^) | 29.6 (±6.2) | 31.3 (±5.5) | <0.0001*** |  |
| SBP (mmHg) | 126.2 (±16.3) | 130.3 (±16.5) | <0.0001 |  |
| DBP (mmHg) | 79.9 (±12.0) | 83.8 (±10.4) | <0.001 |  |
| Tot-Chol (mg/dl) | 194.3 (±37.9) | 202.4 (± 36.7) | 0.154 |  |
| HDL-Chol (mg/dl) | 51.6 (±14.1) | 45.5 (±9.3) | <0.0001 |  |
| LDL-Chol (mg/dl) | 122.3 (±31.7) | 133.3 (±31.6) | 0.360 |  |
| Triglycerides (mg/dl) | 116.2 (±66.6) | 142.3 (±76.0) | <0.0001 |  |
| UA (mg/dl) | 4.81 (±1.25) | 5.29 (±1.24) | <0.0001 |  |
| UHR (%) | 10.12 (±4.71) | 12.38 (±4.88) | <0.0001 |  |
| FPG (mg/dl) | 87.6 (±6.9) | 98.7 (±11.6) | <0.0001 |  |
| 2-h PG (mg/dl) | 105.5 (±18.5) | 141.7 (±28.3) | <0.0001 |  |
| FPI (U/l) | 12.5 (±8.3) | 16.3 (±8.5) | <0.0001 |  |
| HbA1c mmol/mol (%) | 36 ±4 (5.40±0.30) | 38±4 (5.61±0.35) | <0.0001 |  |
| M Clamp | 5.33 (±3.07) | 4.00 (±2.48) | <0.0001 |  |
| Hypolipidemic Teraphy (%) | 1.2 | 5.0 | 0.039 |  |
| Hypotensive Therapy % | 14.7 | 20.0 | 0.258 |  |
| Diuretics (%) | 0.8 | 3.8 | 0.059 |  |
| ARBs (%) | 4.4 | 7.5 | 0.268 |  |
| Smokers %  Current  Ex | 96  67  33 | 28  64  36 | <0.0001  0.655  0.389 |  |

The data are presented as means ± SD for continuous variables and number (percentages) for dichotomous variables. Comparisons were performed using a general linear model with post hoc Bonferroni correction for multiple comparisons and by the χ^2^ test for categorical variables. P values refer to results after analyses with adjustment for age, gender, and BMI. *P values refer to results after analyses with adjustment for age and BMI. ** P values refer to results after analyses with adjustment for gender and BMI. *** P values refer to results after analyses with adjustment for age and gender. BMI, body mass index; FPG, Fasting Plasma Gucose; FPI, Fasting Plasma insulin; HbA1c, Hemoglobin A1c; HDL-Chol, high density lipoprotein; HOMA-IR, the homeotasis model assessment index of insulin resistance; LDL-Chol, low density lipoprotein; 2-h PG, 2h-plasma glucose; Uric acid, UA; uric acid-to- HDL-Chol ratio, UHR; Tot-Chol, Total cholesterol.
