# Supplementary figures and images for: Serum uric acid to HDL-Chol ratio (UHR) is associated with insulin resistance/sensitivity in individuals without diabetes
Source: Acta Diabetol. 2025 Aug 27;63(1):87–95. doi: 10.1007/s00592-025-02576-2 (PMC12847180; doi:10.1007/s00592-025-02576-2)

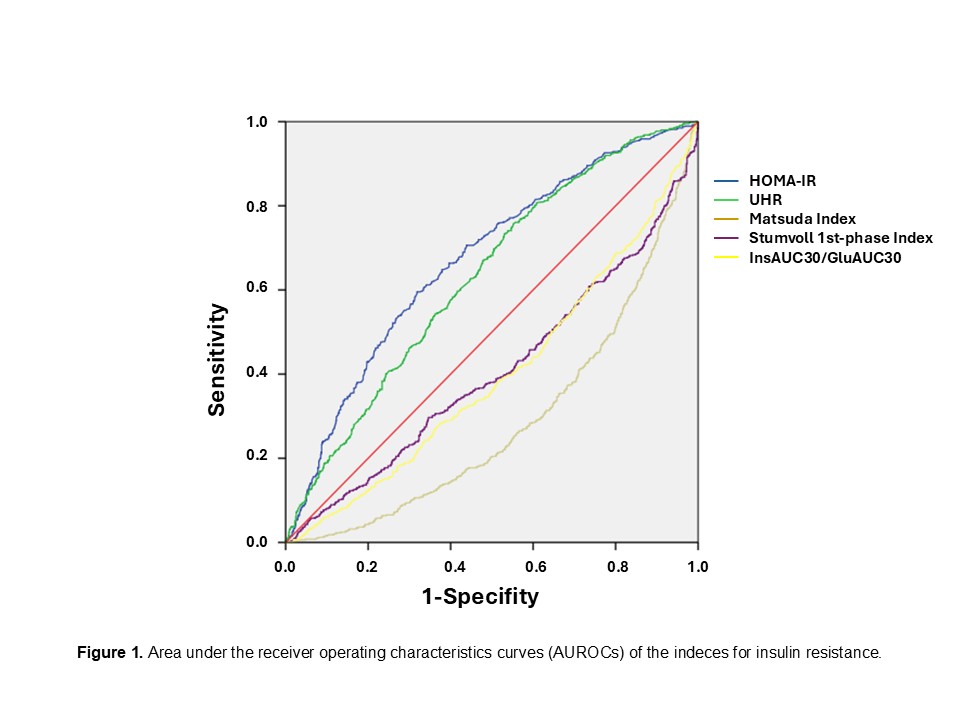

Supplement: Supplementary file 3 — Supplementary Material 3 [file 592_2025_2576_MOESM3_ESM.jpg]
